# Supplementary material for: The Association Between Chronic Obstructive Pulmonary Disease (COPD) and Migraine: Systematic Review and Meta-Analysis
Source: J Clin Med. 2024 Nov 18;13(22):6944. doi: 10.3390/jcm13226944 (PMC11594420; doi:10.3390/jcm13226944)
Supplement: Supplementary file 1 [file jcm-13-06944-s001.zip › jcm-3259160-supplementary.pdf]

Table S1. Quality assessment of the included studies

**Joanna Briggs Institute (JBI) checklist for Case-Control studies**										
**Study**	**Were the groups comparable other than the presence of disease in cases or the absence of disease in controls?**	**Were cases and controls matched appropriately?**	**Were the same criteria used for the identification of cases and controls**	**Was exposure measured in a standard, valid and reliable way?**	**Was exposure measured in the same way for cases and controls?**	**Were confounding factors identified?**	**Were strategies to deal with confounding factors stated?**	**Were outcomes assessed in a standard, valid and reliable way for cases and controls?**	**Wase the exposure period of interest long enough to be meaningful**	**Was appropriate statistical analysis used?**
Davey et al., 2002 [20]	✓	✓	✓	✓	✓	✗	✗	✓	~	✓
**Joanna Briggs Institute (JBI) checklist for Cross-sectional studies**										
**Study**	**Was the sample frame appropriate to address the target population?**	**Were study participants sampled in an appropriate way?**	**Was the sample size adequate?**	**Were the study subjects and the settings described in detail?**	**Was the data analysis conducted with sufficient coverage of the identified sample?**	**Were valid methods used for the identification of the condition?**	**Was the condition measured in a standard, reliable way for all participants?**	**Was there appropriate statistical analysis?**	**Was the response rate adequate, and if not, was the low response rate managed appropriately? Overall**	**NA**
Miguel-Díez et al., 2018 [19]	✓	✓	✓	✓	✓	✗	~	✓	✓	

Minen et al., 2019 [17]	✓	✓	✓	✓	✓	✓	✓	✓	~	
Buse et al., 2010 [16]	✓	✓	✓	✓	✓	✓	✓	✓	✓	
Wang et al., 2016 [18]	✓	✓	✓	✓	✓	✓	✓	✓	✓	

Legend: ✓, low risk of bias; ✗, high risk of bias; ~, and moderate risk of bias/some concern
